# A Facile Way to Fabricate High-Performance Solution-Processed n-MoS_2_/p-MoS_2_ Bilayer Photodetectors

**DOI:** 10.1186/s11671-015-1161-3

**Published:** 2015-11-25

**Authors:** Jian Ye, Xueliang Li, Jianjun Zhao, Xuelan Mei, Qian Li

**Affiliations:** Department of Chemistry and Environmental Engineering, Bengbu College, Bengbu, Anhui 233030 China; School of Chemistry and Chemical Engineering, Hefei University of Technology, Hefei, Anhui 230009 China

**Keywords:** Liquid exfoliation method, Partially oxided MoS_2_, Photodetectors

## Abstract

Two-dimensional (2D) material has many advantages including high carrier mobilities and conductivity, high optical transparency, excellent mechanical flexibility, and chemical stability, which made 2D material an ideal material for various optoelectronic devices. Here, we developed a facile method of preparing MoS_2_ nanosheets followed by a facile liquid exfoliation method via ethyl cellulose-assisted doping and utilizing a plasma-induced p-doping approach to generate *t* effectively the partially oxided MoS_2_ (p-MoS_2_) nanosheets from the pristine n-type nanosheets. Moreover, an n-p junction type MoS_2_ photodetector device with the built-in potentials to separate the photogenerated charges is able to significantly improved visible light response. We have fabricated photodetector devices consisting of a vertically stacked indium tin oxide (ITO)/pristine n-type MoS_2_ nanosheets/p-MoS_2_/Ag structure, which exhibit reasonably good performance illumination, as well as high current values in the range of visible wavelength from 350 to 600 nm. We believe that this work provides important scientific insights for photoelectric response properties of emerging atomically layered 2D materials for photovoltaic and other optoelectronic applications.

## Background

Over the last decade, two-dimensional (2D) nanomaterials have drawn great attention because of their unique structures, large natural abundance, and distinctive properties compared to their bulk forms, and a broad range of applications in catalysis, electronics, energy-storage devices, optoelectronics, and so on [1–11]. In particular, the semiconducting layered transition metal dichalcogenides (LTMDs, e.g., WSe_2_, WS_2_, and MoS_2_) have gained significant interest on optoelectronics due to their direct bandgaps, possessing intriguing optical properties suitable for optoelectronic applications in light-emitting diodes and photovoltaics [12–14]. Usually, LTMDs have a unique 2D X–M–X structure in which the transition metal atom layer is sandwiched between two close-packed chalcogen atom layers [1, 2, 15–17].

As a prototypical compound of LTMDs, MoS_2_ has been extensively studied. Bulk MoS_2_ is a typical semiconductor with an indirect bandgap. Expectedly, monolayer MoS_2_ transistors have been demonstrated with on/off ratios of 10^8^ and ultralow standby power dissipation [17–19]. However, to realize the highly efficient optoelectronic devices based on MoS_2_, it is also important to develop a strategy to prepare ultrathin MoS_2_ nanosheets and tune the bandgaps with facile process. Several methods, such as mechanical exfoliation (the so-called Scotch tape method), liquid exfoliation, colloidal synthesis, chemical vapor deposition, chemical exfoliation, and electrochemical exfoliation have been developed to prepare ultrathin MoS_2_ nanosheets [2, 20–30]. Among these methods, liquid exfoliation not only produces novel materials with the same composition yet dramatically changed electrical properties but also provides a facile way to prepare thin-layer nanosheets, which offers novel opportunities in the optoelectronics applications [17, 31–34].

In this work, we report that a novel liquid exfoliation method via ethyl cellulose-assisted doping can prepare an excellent thin MoS_2_ nanosheets and very effective method to generate the partially oxidized MoS_2_ (p-MoS_2_) nanosheets from the pristine n-type nanosheets. Moreover, an n-p junction type MoS_2_ photodetector device with the built-in potentials to separate the photogenerated charges can result in significantly improved visible light response. We have fabricated photodetector devices consisting of a vertically stacked indium tin oxide (ITO)/pristine n-type MoS_2_ nanosheets/p-MoS_2_/Ag structure, which exhibit reasonably good performance illumination, as well as high current values in the range of visible wavelength from 350 to 600 nm. This work provides important scientific insights for leveraging unique optoelectronic properties of 2D materials for photodetector applications.

## Methods

### Material Synthesis

Molybdenum disulfide (MoS_2_) nanosheets were synthesized by liquid ultrasound exfoliation as reported in the literature [35, 36]. Typically, MoS_2_ power (0.25 g, Aladdin) was dispersed in ethyl cellulose (EC) isopropanol solution (1 % *w*/*v* dispersion, 100 ml) in a SEBC bottle. The dispersion was sonicated for 24 h at 60 W in water bath. The resulting dispersion was centrifuged (Desktop High-speed Refrigerated Centrifuge Model TGL-16) at 5000 rpm for 15 min, and then the supernatant liquid was directly collected. Deionized water was mixed with the supernatant liquid (3:4 weight ratio) and subsequently centrifuged at 7500 rpm for 10 min. Whereafter, the lower precipitation was collected and dried. The resulting precipitation was redispersed in ethanol (10 mg/ml). NaCl aqueous solution (0.04 g/ml) was mixed with the redispersion (9:16 weight ratio) and centrifuged at 5000 rpm for 8 min, discarding the supernatant. To debride any residual salt, the resulting MoS_2_ precipitation was washed with deionized water and collected by vacuum filtration (0.45 μm filter paper). Finally, the MoS_2_ nanosheet product was dried as a fine black powder. The final MoS_2_ nanosheets were defined as n-MoS_2_. For the preparation of p-MoS_2_ nanosheets, the n-MoS_2_ powder was taken a UV-ozone plasma treatment for 40 min to completely change to p-MoS_2_ nanosheets.

### Characterizations

TEM images were taken by a FEI TECNAI G2 F20-TWIN TEM. Raman spectra were recorded on inVia Raman microscope. XPS and UPS measurements were conducted using an ESCALAB 250Xi (Thermo) system. X-ray diffraction (XRD) patterns of the MoS_2_ was carried out on a Bruker D8 Focus X-ray diffractometer operating at 30 kV and 20 mA with a copper target (λ= 1.54 Å) and at a scanning rate of 1°/min.

### Photodetector Device Fabrication

All devices were fabricated on pre-treatment ITO glass substrates [37] (sheet resistance <10 Ωsq^−1^, ShenZhen NanBo Display Technology Co., Ltd.); cleaned sequentially using sonication in acetone, detergent, deionized water, and isopropanol; and then dried under a nitrogen stream, followed by ultraviolet light irradiation. Then, the n-MoS_2_ nanosheets (10 mg/ml, in isopropanol) spin coated with 2000 rpm and thermally annealed at 150 °C for 15 min receive a thickness of 80 nm. Thereafter, the p-MoS_2_ nanosheets (15 mg/ml, in isopropanol) was spin coated on n-MoS_2_ nanosheets layer, followed by thermal annealing at 150 °C for 10 min in atmospheric environment. Eventually, Argentum Ag (150 nm) was deposited over the p-MoS_2_ nanosheets layer by thermal evaporation under a vacuum of 6 × 10^−6^ Torr to accomplish the device fabrication. The effective area of one cell was ~1 cm^2^. The photocurrent-voltage curves and I-T curves were measured with a Keithley 2400 source meter and a 150-W Xe lamp light source. The dark current-voltage curves were measured by Keithley 2400 source meter under dark. All the measurements were performed under ambient atmosphere at room temperature. The incident photo-to-electron conversion efficiency spectrum (IPCE) were detected under monochromatic illumination (Oriel Cornerstone 260 1/4 m monochromator equipped with Oriel 70613NS QTH lamp), and the calibration of the incident light was performed with a monocrystalline silicon diode.

## Results and Discussion

**Fig. 1:**
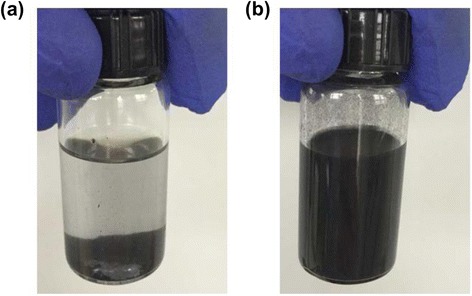
The images are camera pictures of **a** pristine MoS_2_ and **b** MoS_2_ nanosheets dispersion

The equal concentration of pristine MoS_2_ and MoS_2_ nanosheets after the liquid ultrasound exfoliation solution (10 mg/ml) was treated with ultrasound in ethanol for 30 min, respectively. The detailed process is demonstrated in experimental section. The photographs of pristine MoS_2_ and MoS_2_ nanosheets isopropanol dispersion solutions after ultrasound treatment are shown in Fig. 1. After storing for 48 h, humorous aggregation can be observed in pristine MoS_2_ solution (Fig. 1a) and evident MoS_2_ particles adhere to the sidewall. In contrast, the MoS_2_ nanosheets after the liquid ultrasound exfoliation solution show a highly uniform and homogeneous suspension solution (Fig. 1b), indicating the successful preparation of MoS_2_ nanosheets with the good dispensability.

**Fig. 2:**
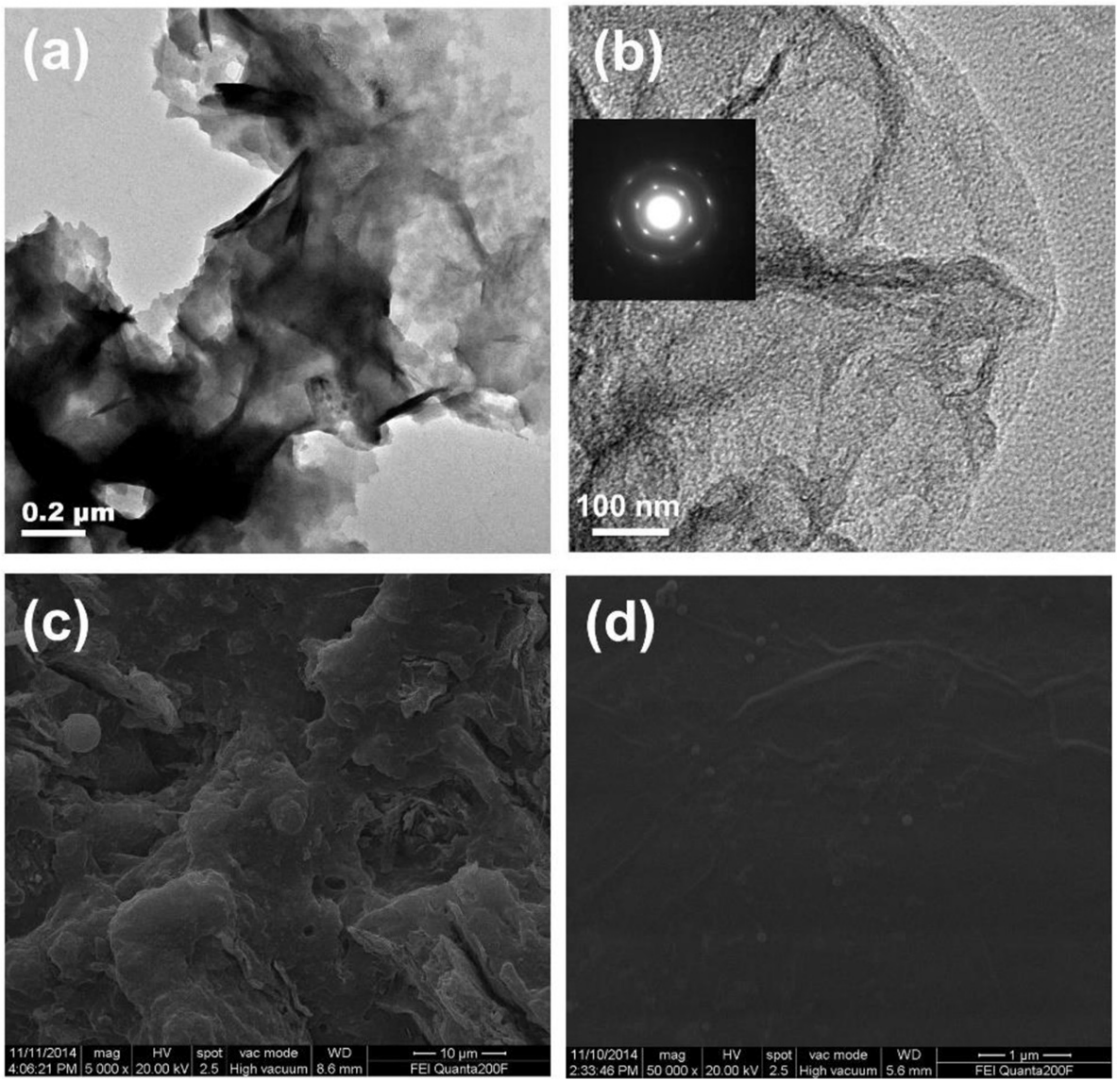
The transmission electron microscopy (TEM) images of **a** pristine MoS_2_ and **b** MoS_2_ nanosheets films on glass substrate, and the *inset* is selected area electron diffraction (SAED) pattern of the MoS_2_ nanosheets. The scanning electron microscopy (SEM) images of **c** pristine MoS_2_ and **d** MoS_2_ nanosheets films on glass substrate

In order to verify the degree of dispersion of exfoliated MoS_2_ nanosheets by ethyl cellulose ethanol solution via liquid ultrasound exfoliation, transmission electron microscopy (TEM) and scanning electron microscopy (SEM) were performed (Fig. 2). For comparison, the morphologies of the pristine MoS_2_ nanosheets prepared by 150 °C thermal annealing for 10 min were also determined. All of samples were spin-coated on ITO and tested in the same testing conditions. Figure 2a shows a rough morphology of the pristine MoS_2_, and clearly stacked MoS_2_ can be seen. However, Fig. 2b displays an individual MoS_2_ sheet with six spot pattern in the selected-area electron diffraction (SAED) of MoS_2_, suggesting that MoS_2_ is scattered as individual MoS_2_ nanosheet [38, 39]. Also, the severe aggregation of the pristine MoS_2_ can be observed in SEM images (Fig. 2c), intriguingly, after being treated by ethyl cellulose ethanol solution via liquid ultrasound exfoliation, MoS_2_ nanosheets can fully cover and tightly attach on the ITO substrate with a quite smooth surface morphology (Fig. 2d).

**Fig. 3:**
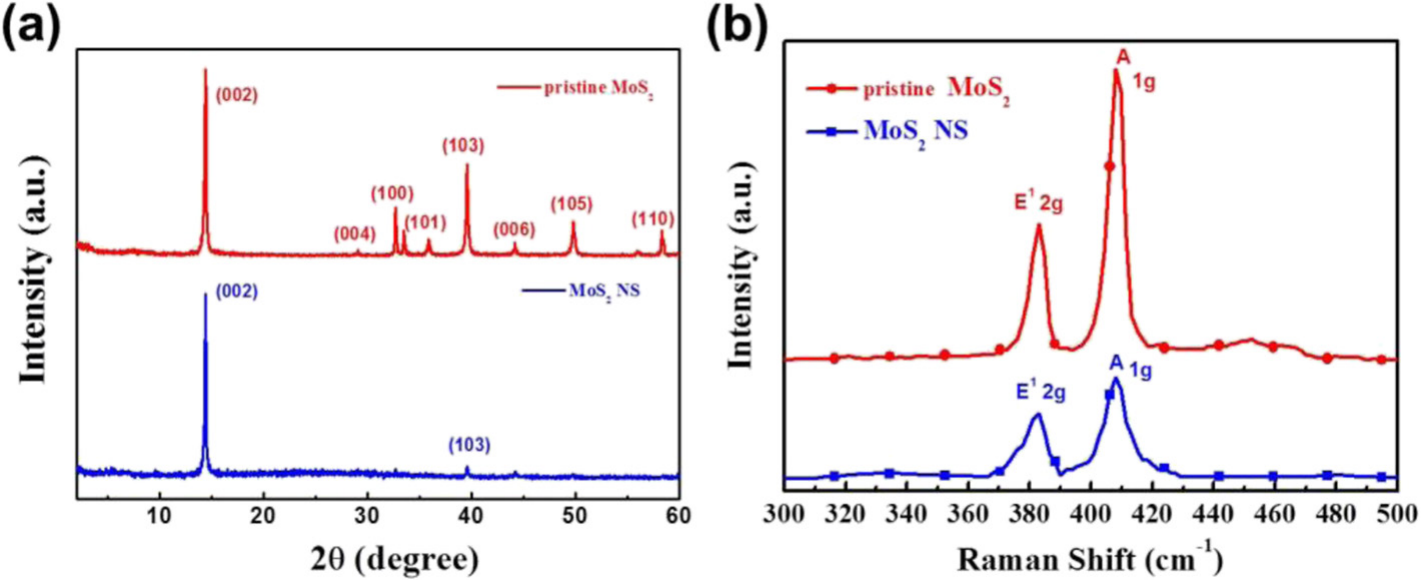
**a** XRD patterns of the MoS_2_ films on glass substrate. **b** Raman spectrum of MoS_2_ films on glass substrate

To further verify morphology results, the XRD patterns of pristine and exfoliated MoS_2_ nanosheets (Fig. 3a) only the peaks of (103) and (002) plane remain after liquid exfoliation which confirms that the MoS_2_ nanosheets were successfully striped [40, 41]. Moreover, the disappearance of other peaks could prove that ultrathin MoS_2_ nanosheets are tightly deposited on the ITO glass with preferred ductility. The Raman spectrum can once again prove the exfoliation of MoS_2_ nanosheets. The two peaks (1 and 2 g) between 360 and 430 cm^−1^ are the main peak of MoS_2_ [42–44]. After liquid exfoliation, the obvious decrease of the intensity of the two peaks was observed.

**Fig. 4:**
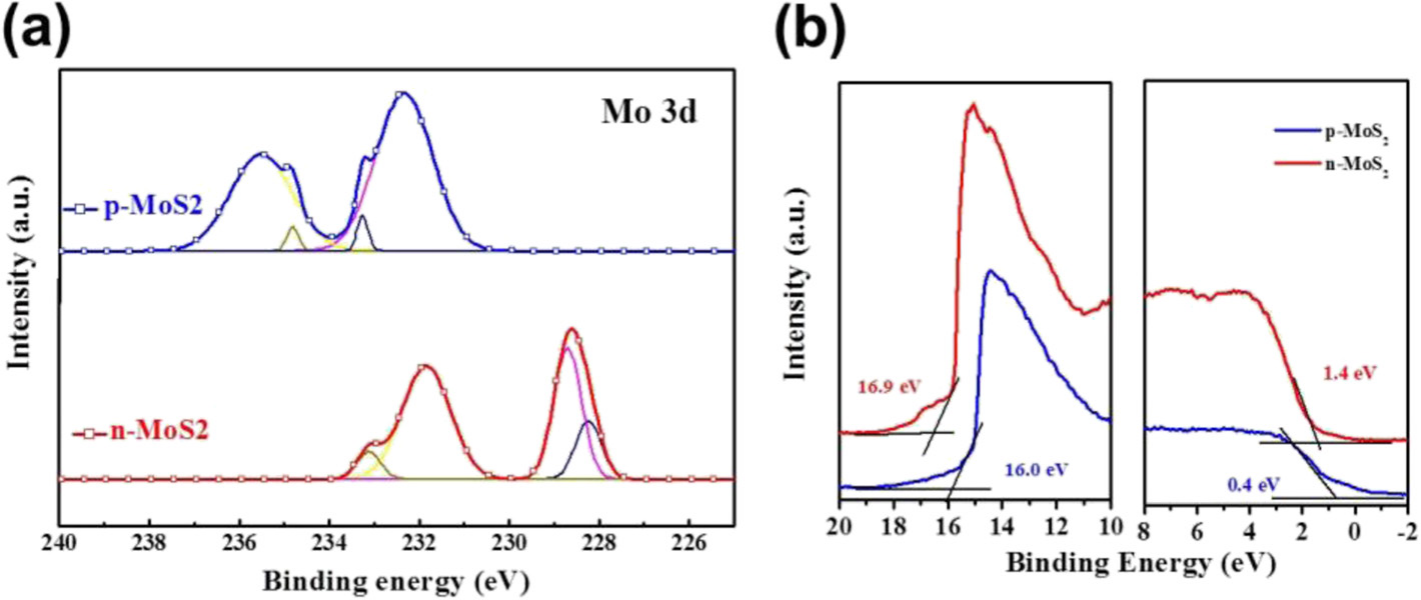
**a** Mo 3D region and of X-ray photoelectron spectroscopy (XPS) profiles of MoS_2_ nanosheets with or without plasma treatment. **b** The ultraviolet photoelectron spectroscopy (UPS) spectra of MoS_2_ nanosheets with or without plasma treatment

It is well known that the MoS_2_ nanosheets are n-type semiconductor materials and several researches have been reported that MoS_2_ could be changed as a p-type semiconductor material with a relative high work function after UV-ozone plasma treatment. Thus, the properties of MoS_2_ nanosheets with or without the UV-ozone plasma treatment were also investigated. Figure 4a is the X-ray photoelectron spectroscopy (XPS) profile of n-MoS_2_ nanosheets (without plasma treatment) and p-MoS_2_ nanosheets (with plasma treatment). The Mo 3D spectra of pristine MoS_2_ nanosheets demonstrate outstanding Mo^4+^3d_5/2_ and Mo^4+^3d_3/2_ bands at 228.7 and 231.5 eV, in agreement with the other works for n-MoS_2_ nanosheets. However, the two strong peaks have a notable shift to 235.3 and 232.5 eV, respectively, which is similar with the spectra of MoO_3_ [45, 46]. Therefore, it proved that n-MoS_2_ nanosheets can be successfully oxidized to p-type materials after UV-ozone plasma treatment. Since the MoS_2_ layer is very thin via the spin-coating method, it is important to analyze the bilayer junction existing at the interface of n-MoS_2_/p-MoS_2_. To gain insight into the electronic structures of the n-MoS_2_/p-MoS_2_ bilayer junction, we have performed the UPS analysis. The work function was calculated through the difference between the cutoff of the highest binding energy and the photon energy of the exciting radiation. The valence band (VB) can be calculated from the cutoff from the lowest binding energy. As shown in Fig. 4b, after UV-ozone plasma treatment, the work function of the MoS_2_ nanosheets has increased from 4.3 to 5.2 eV. The energy difference between the Fermi level and valence band maximum is decreased from 1.4 to 0.4 eV, demonstrating the n-type MoS_2_ nanosheets change to p-type MoS_2_ nanosheets [47].

**Fig. 5:**
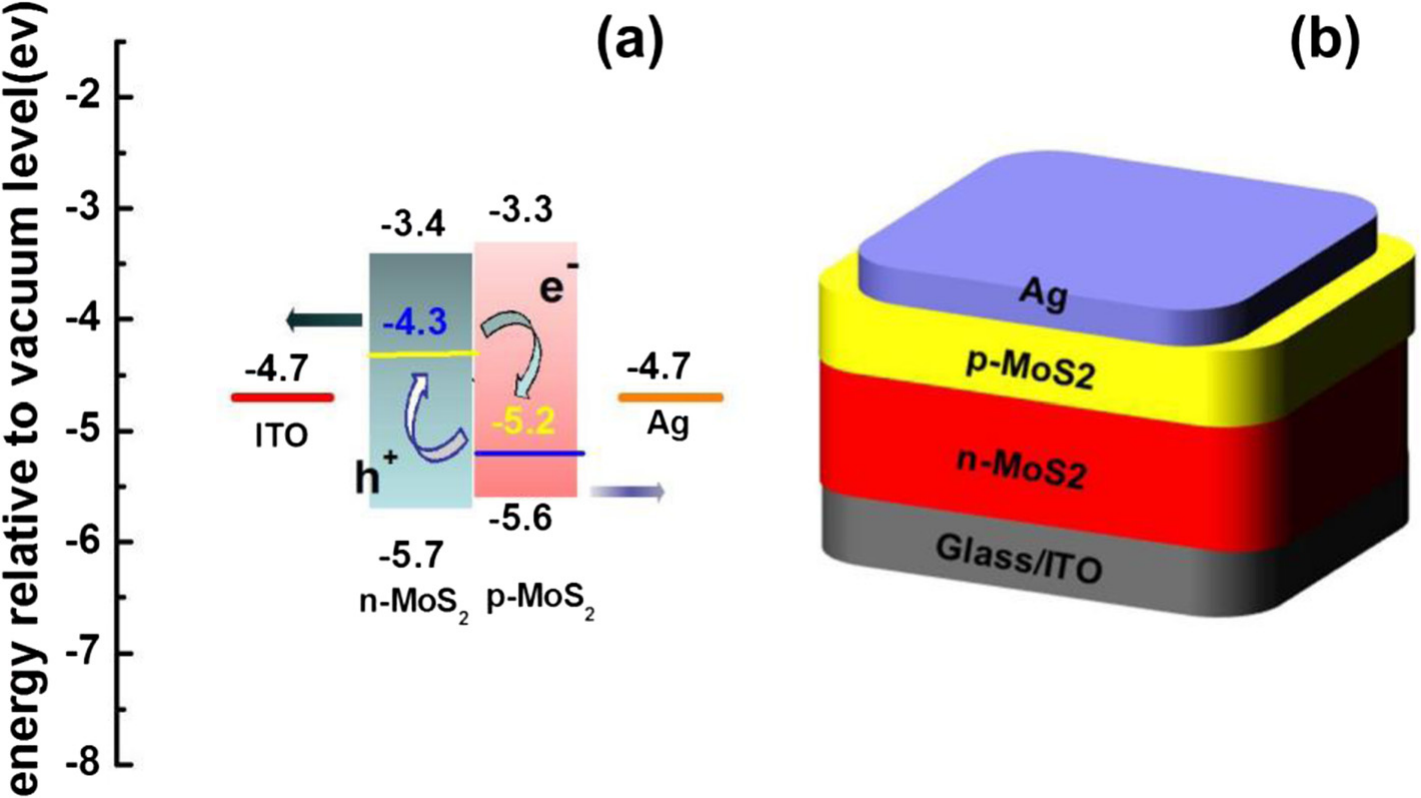
**a** The schematic energy diagram of the MoS2 photodetector **b** the structure of MoS2 photodetector device

**Fig. 6:**
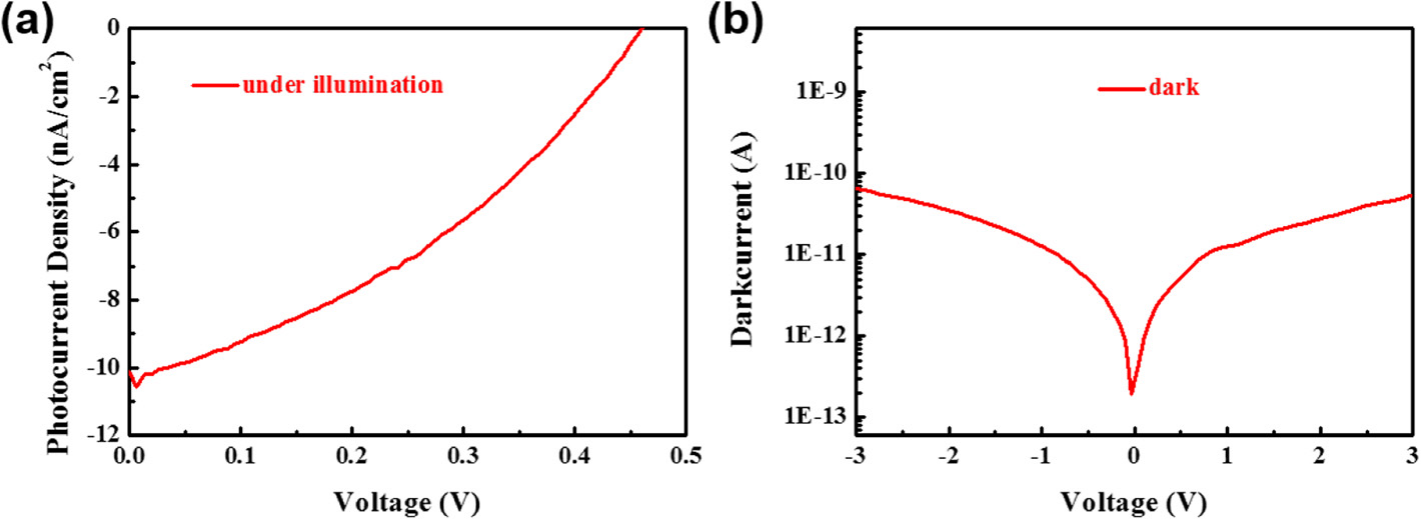
Current-voltage curves of the device **a** under a 150-W Xe lamp light source illumination and **b** in dark

**Fig. 7:**
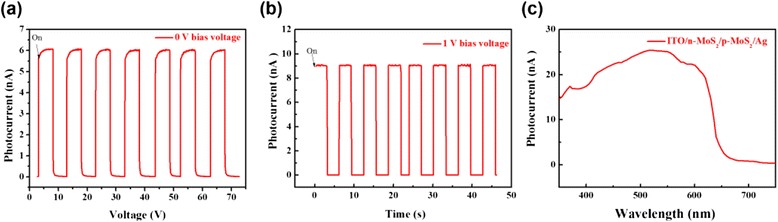
**a** The output signal of photocurrent under alternating light on and light off, where the entire device was illuminated by a 150-W Xe lamp irradiation. Photoresponse of MoS_2_-based photodetector at a 0-V DC bias voltage. **b** Photoresponse of MoS_2_-based photodetector at 1-V DC bias voltage. **c** The spectral photoresponse vs. wavelength, showing a broad photoresponse range from 350 to 650 nm, which is, the absorption spectrum of the nanohybrid covers the whole energy range of visible light

On the basis of the above results, we have constructed an energy diagram showing the band bending behavior at the n-MoS_2_/p-MoS_2_ bilayer junction interface, as shown in Fig. 5a. The n-MoS_2_/p-MoS_2_ bilayer junction with a built-in potential promises an excellent photodetector performance with a ITO/n-MoS_2_/p-MoS_2_/Ag device structure (Fig. 5b) which will be discussed later. The photocurrent-voltage curves and the photocurrent-voltage were measured with the Keithley 2400 source meter. As shown in Fig. 6a, b, the device shows the photovoltaic response under a 150-W Xe lamp light source illumination. The result shows the device have a p-n junction inside. In order to understand the photoelectric response properties in more detail and detect potential application in photoelectronic fields, we have performed further experiments of photodetector at a 1-V DC bias as shown in Fig. 7a, b. As seen from Fig. 7a, b, the photocurrent increases at an applied dc bias voltage of 0 and 1 V. Moreover, the photoresponse is steady, prompt, and reproducible during repeated on/off cycles of visible light illumination. More importantly, the n-MoS_2_/p-MoS_2_ bilayer junction-based device shows a very broad photoelectric response range from 350 to 600 nm, as shown in Fig. 7c, and therefore, the n-MoS_2/_p-MoS_2_ bilayer junction can harvest nearly the whole energy range of visible light.

## Conclusions

We have demonstrated a high-quality n-MoS_2_/p-MoS_2_ bilayer junction-based device to achieve the high performance photoresponse which can harvest nearly the whole energy range of visible light. Excellent, thin exfoliated MoS_2_ nanosheets are realized by a facile liquid exfoliation, changing the n-type MoS_2_ nanosheets to p-type MoS_2_ nanosheets via a simple plasma treatment. This work shows that thin MoS_2_ nanosheets can be fully integrated into the photodetector manufacturing process, which holds promise for realizing 2D materials in a variety of optical electronic and optical devices.
